# *KCNJ11*, *ABCC8* and *TCF7L2* polymorphisms and the response to sulfonylurea treatment in patients with type 2 diabetes: a bioinformatics assessment

**DOI:** 10.1186/s12881-017-0422-7

**Published:** 2017-06-06

**Authors:** Jingwen Song, Yunzhong Yang, Franck Mauvais-Jarvis, Yu-Ping Wang, Tianhua Niu

**Affiliations:** 1 Department of Global Biostatistics and Data Science, Tulane University School of Public Health and Tropical Medicine, New Orleans, LA 70112 USA; 2 Division of Endocrinology and Metabolism, Department of Medicine, Tulane University Health Sciences Center, New Orleans, LA 70112 USA; 3 Department of Biomedical Engineering, Tulane University School of Science and Engineering, New Orleans, LA 70118 USA

**Keywords:** Sulfonylurea, Type 2 diabetes, Pharmacogenetics, *ABCC8*, *KCNJ11*, *TCF7L2*, Single nucleotide polymorphism, Bioinformatics, *In silico*

## Abstract

**Background:**

Type 2 diabetes (T2D) is a worldwide epidemic with considerable health and economic consequences. Sulfonylureas are widely used drugs for the treatment of patients with T2D. *KCNJ11* and *ABCC8* encode the K_ir_6.2 (pore-forming subunit) and SUR1 (regulatory subunit that binds to sulfonylurea) of pancreatic β cell K_ATP_ channel respectively with a critical role in insulin secretion and glucose homeostasis. *TCF7L2* encodes a transcription factor expressed in pancreatic β cells that regulates insulin production and processing. Because mutations of these genes could affect insulin secretion stimulated by sulfonylureas, the aim of this study is to assess associations between molecular variants of *KCNJ11*, *ABCC8* and *TCF7L2* genes and response to sulfonylurea treatment and to predict their potential functional effects.

**Methods:**

Based on a comprehensive literature search, we found 13 pharmacogenetic studies showing that single nucleotide polymorphisms (SNPs) located in *KCNJ11*: rs5219 (E23K), *ABCC8*: rs757110 (A1369S), rs1799854 (intron 15, exon 16 -3C/T), rs1799859 (R1273R), and *TCF7L2*: rs7903146 (intron 4) were significantly associated with responses to sulfonylureas. For *in silico* bioinformatics analysis, SIFT, PolyPhen-2, PANTHER, MutPred, and SNPs3D were applied for functional predictions of 36 coding (*KCNJ11*: 10, *ABCC8*: 24, and *TCF7L2*: 2; all are missense), and HaploReg v4.1, RegulomeDB, and Ensembl’s VEP were used to predict functions of 7 non-coding (*KCNJ11*: 1, *ABCC8*: 1, and *TCF7L2*: 5) SNPs, respectively.

**Results:**

Based on various *in silico* tools, 8 *KCNJ11* missense SNPs, 23 *ABCC8* missense SNPs, and 2 *TCF7L2* missense SNPs could affect protein functions. Of them, previous studies showed that mutant alleles of 4 *KCNJ11* missense SNPs and 5 *ABCC8* missense SNPs can be successfully rescued by sulfonylurea treatments. Further, 3 *TCF7L2* non-coding SNPs (rs7903146, rs11196205 and rs12255372), can change motif(s) based on HaploReg v4.1 and are predicted as risk factors by Ensembl’s VEP.

**Conclusions:**

Our study indicates that a personalized medicine approach by tailoring sulfonylurea therapy of T2D patients according to their genotypes of *KCNJ11*, *ABCC8*, and *TCF7L2* could attain an optimal treatment efficacy.

## Background

The prevalence of diabetes is increasing at a fast rate, which was 6.4% (285 million) among adults aged 20–79 years in 2010, and will increase to 7.7% (438 million) by 2030 [1]. Among all diabetic cases, approximately 90% are patients with type 2 diabetes (T2D), which is associated with a number of microvascular complications including retinopathy, nephropathy, neuropathy, as well as macrovascular complications [2]. T2D is caused by a plethora of lifestyle and genetic factors [3, 4]. Current therapies for T2D include life-style modifications and use of oral antidiabetic drugs, with sulfonylurea being one of the most frequently used one [5]. There are a number of different sulfonylurea treatments for T2D patients, among which the commonly used ones are gliclazide, glibenclamide, glimepiride and glipizide [6].

**Fig. 1:**
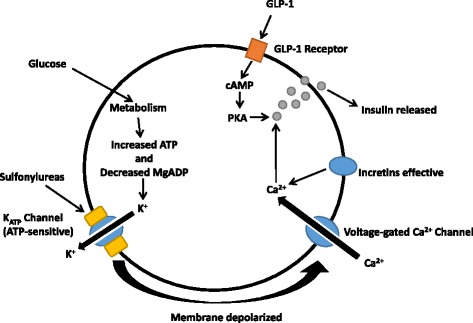
A schematic representation of the pancreatic β cell illustrating the molecular model for insulin secretion mediated by K_ATP_ channel comprising KCNJ11 and ABCC8 subunits in sulfonylurea treatment

Sulfonylurea promotes insulin secretion from the pancreatic β cells of the pancreas in a glucose-independent manner by binding to ATP-sensitive K^+^ (K_ATP_) channel on the cell membrane of pancreatic β cells. K_ATP_ channel is a hetero-octamer comprising the inward-rectifier potassium ion channels K_ir_6.x (i.e., K_ir_6.1 and K_ir_6.2) that form the pore, and sulfonylurea receptors (SUR; i.e., SUR1, SUR2A, and SUR2B) that regulate the opening and closing of its associated K_ir_6.x potassium channel, as SUR is sensitive to ATP and ADP levels. The binding of sulfonylureas to the corresponding receptors could lead to an efflux of intracellular potassium, hyperpolarization of the β cell membrane, and the opening of voltage-gated calcium channels, which result in an increased secretion of insulin to circulation (Fig. 1).

The pancreatic β cell K_ATP_ channel consists of four pore-forming subunits of the inwardly rectifying potassium channel Kir6.2 and four regulatory subunits of the SUR1 [7–9]. When blood glucose concentrations rise, an increase in glucose metabolism results in a change of ADP/ATP ratio, which leads to a closing of K_ATP_ channel. The respective genes encoding K_ir_6.2 and SUR1, i.e., *KCNJ11* and *ABCC8*, are located next to each other on human chromosome 11p15.15. Mutations in *KCNJ11* or *ABCC8* genes could decrease or abolish the metabolic sensitivity of β cell K_ATP_ channel function, leading to a constant depolarization of the cell membrane and a persistent insulin secretion even at very low plasma glucose concentrations [10]. E.g., single nucleotide polymorphism (SNP) E23K (i.e., rs5219) of *KCNJ11* gene is associated with T2D risk (reviewed in [11]), is shown to result in a decrease or loss of sensitivity of K_ATP_ channel to the inhibitory effect of ATP [12] and/or an enhancement of activation by free fatty acids [13]. Further, mutations in *ABCC8* gene could cause hyperinsulinemic hypoglycemia [10]. The β cell K_ATP_ channel can be pharmacologically regulated by sulfonylureas, which function by binding to and closing the K_ATP_ channel [14] that leads to membrane depolarization, which subsequently results in an activation of voltage-dependent calcium channels causing an influx of calcium, which then triggers insulin granule exocytosis.

*TCF7L2* encodes a member of the T-cell factor (TCF) transcription factor that plays a critical role in Wnt signaling pathway [15], which is shown to be involved in β cell dysfunction in T2D [16]. TCF7L2 is a member of the TCF-lymphocyte enhancer factor (LEF) protein family [17], and the bipartite transcription factor β-catenin/TCF-LEF serves as an effector of cAMP-dependent protein kinase A (PKA) signaling to mediate the physiological effects of peptide hormones including glucagon-like peptide-1 (GLP-1), which utilizes cAMP as a second messenger [18, 19]. *TCF7L2* gene SNPs are strongly associated with a higher risk of T2D development [15], which could be mediated by their influences on blood glucose homeostasis [20].

**Fig. 2:**
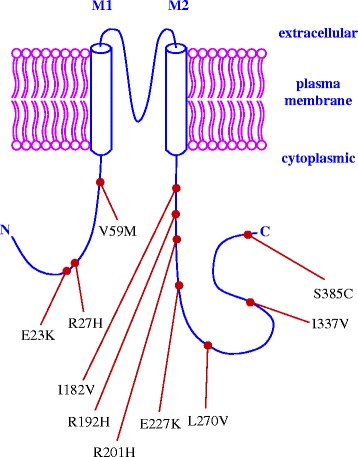
A schematic representation of 10 *KCNJ11* missense SNPs in the protein product. Each Kir6.2 subunit (i.e., KCNJ11 protein product) contains two transmembrane domains, M1 and M2. Between M1 and M2, there is a pore-forming loop that creates the core of the K^+^ channel

**Fig. 3:**
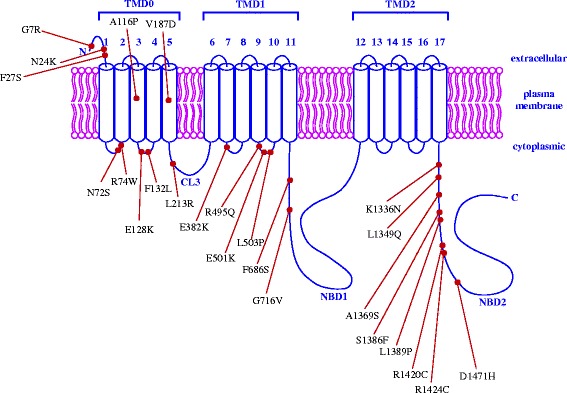
A schematic representation of 24 *ABCC8* missense SNPs in the protein product. Each SUR1 subunit (i.e., ABCC8 protein product) contains 17 transmembrane helices, which are arranged in three transmembrane domains, i.e., TMD0, TMD1, and TMD2, respectively. The large cytosolic loop between TMD0 and TMD1 is called cytosolic loops 3 (CL3). The large cytosolic domains following TMD1 and TMD2 contain nucleotide-binding domain 1 (NBD1) and NBD2, respectively

**Fig. 4:**
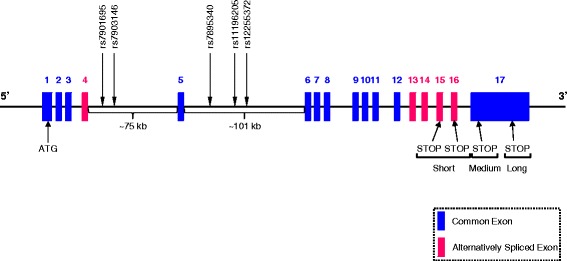
A schematic representation of 5 *TCF7L2* non-coding SNPs in the gene structure. The start and stop codons are indicated by “ATG” (in exon 1) and “STOP” (in exons 15, 16, and 17), respectively. Because of alternative splicing, 3 groups of protein isoforms (i.e., short-, medium-, and large-length isoforms) can be generated by using different stop codons, which are indicated by “Short”, “Medium”, and “Long”, respectively

Sulfonylureas show considerable inter-individual variations in the hypoglycemic response, with approximately 10–20% of patients having a less than 20 mg/dl reduction in fasting plasma glucose (FPG) following the initiation of sulfonylurea therapy (called primary sulfonylurea failure) [21]. Further, about 50–60% of patients will initially have a greater than 30 mg/dl reduction in FPG, but will fail to reach the desired glycemic treatment goals [21]. In contrast, some T2D patients could have higher risks of mild or severe hypoglycemia in response to sulfonylurea treatment [22–24]. Molecular variants of sulfonylurea drug target genes *KCNJ11*, *ABCC8*, and *TCF7L2* could lead to different responses to sulfonylurea therapy in T2D patients. Therefore, their impacts need to be carefully evaluated. The primary objective of this study is to predict functional effects of 36 coding (*KCNJ11*: 10, *ABCC8*: 24, and *TCF7L2*: 2, and all missense) and 7 non-coding (*KCNJ11*: 1, *ABCC8*: 1, and *TCF7L2*: 5) SNPs that were identified from published literatures and MutDB database (http://www.mutdb.org/) by applying a spectrum of *in silico* bioinformatics tools. Each Kir6.2 subunit has two transmembrane domains called M1 and M2, and the pore-forming domain is located between them [25]. The locations of 10 missense SNPs (including the well-studied E23K) in the KCNJ11 protein that comprises 390 amino acids [26] are shown in Fig. 2, respectively. Each SUR1 subunit has three transmembrane domains, i.e., TMD0, TMD1, and TMD3, and two nucleotide binding domains, i.e., NBD1 and NBD2. Between TMD0 and TMD1, there is a cytosolic loop called CL3 [27]. The locations of 24 missense SNPs (including the well-studied A1369S) in the ABCC8 protein that comprises 1581 amino acids [28] are shown in Fig. 3. The human *TCF7L2* gene consists of 17 exons, five of which are alternatively spliced (i.e., exons 4, 13, 14, 15, and 16) and exhibits tissue-specific expression [29]. The differential splicing of *TCF7L2* potentially gives rise to three groups of protein isoforms (i.e., short-, medium-, and large-length isoforms) with highly differential functional properties. These three groups depend on the predicted stop codon usages, which are located in exons 15, 16, 17 [30]. To date, *TCF7L2* intronic SNP, rs7903146, represents the most significant risk variant for T2D [31]. However, four other non-coding SNPs, i.e., rs7901695, rs7895340, rs11196205 and rs12255372, have also been significantly associated with an increased risk of T2D [32] and have been widely studied. The locations of these 5 non-coding SNPs in the gene structure of *TCF7L2* (including the well-studied intronic SNP rs7903146) are illustrated in Fig. 4.

## Methods

### Literature search strategy

Comprehensive electronic literature searches of databases including PubMed, Google Scholar, Cochrane Library, Excerpta Medica Database (EMBASE) were performed up to June 1, 2016 using the following keywords: sulfonylurea, type 2 diabetes, *KCNJ11*, *ABCC8*, and *TCF7L2*. A manual search of the references cited in initially identified articles was also performed. Furthermore, we searched all relevant references of three comprehensive review articles [5, 33, 34]. The search was restricted to English language articles.

### Inclusion and exclusion criteria

Randomized controlled trials and observational studies were eligible for inclusion in the current study. *In vitro* studies, animal studies, letters, reviews, and unrelated articles and duplicates were excluded from this study.

### Data extraction

From each included study, the following data were extracted: first author, publication year, SNP name, gene name, National Center for Biotechnology Information (NCBI) dbSNP (http://www.ncbi.nlm.nih.gov/snp/) ID, study design, study subjects, control source, length of follow-up, and results.

### *In silico* bioinformatics analysis

#### Computational predictions of functional impacts of non-synonymous SNPs (nsSNPs)

Five *in silico* tools were applied: (i) SIFT [35] (http://sift.jcvi.org/), (ii) PolyPhen-2 [36] (http://genetics.bwh.harvard.edu/pph2/), (iii) PANTHER [37] (http://www.pantherdb.org/tools/csnpScore.do), (iv) MutPred [38]) (http://mutpred.mutdb.org/), and (v) SNPs3D [39] (http://www.snps3d.org/).

#### Computational predictions of functional impacts of non-coding SNPs

Three *in silico* tools were applied: (i) HaploReg v4.1 [40, 41] (http://www.broadinstitute.org/mammals/haploreg/haploreg.php), (ii) RegulomeDB [42] (http://regulomedb.org/), and (iii) Ensembl’s VEP [43] (http://www.ensembl.org/Homo_sapiens/Tools/VEP?db=core).

## Results

A total of 17 articles corresponding to 17 independent studies were qualified and subsequently included for evaluating the relationships between *KCNJ11*, *ABCC8* and *TCF7L2* SNPs and response to sulfonylurea in patients with T2D. The detailed characteristics of these 17 studies [44–60] were presented in Table 1. Of them, 13 studies gave positive results, which showed that 1 SNP located in *KCNJ11* rs5219 (E23K) [45–47, 49, 50, 52], 3 SNPs located in *ABCC8*: rs757110 (A1369S) [46, 56], rs1799854 (intron 15, exon 16 -3C/T) [48, 53, 55], rs1799859 (R1273R) [48, 55], and 1 SNP located in *TCF7L2*: rs7903146 (intron 4) [58–60], were significantly associated with responses to sulfonylureas. It is noteworthy that no uniform definition of response to sulfonylurea therapy was used across these 17 independent studies. Javorsky et al. (2012) [50] defined response to sulfonylurea as change in HbA1c level to sulfonylurea at 6-month therapy. Feng et al. (2008) [46] defined response to sulfonylurea as percent decrease in FPG and also FPG at day 57 as < 7.8 mmol/l as well as percent decrease in HbA1c after 8-week sulfonylurea therapy, and Holstein et al. (2009) [47] defined this drug response phenotype as sulfonylurea-induced hypoglycemia, which refers to a symptomatic event requiring treatment with intravenous glucose that was confirmed by a blood glucose measurement of <50 mg/dl. Meirhaeghe et al. (2001) [53] defined response to sulfonylurea as post-treatment fasting insulin, FPG, fasting plasma total cholesterol, and fasting plasma triglyceride concentrations, and Schroner et al. (2011) [59] defined this drug response phenotype as change of HbA1c (%) and changes of FPG for 3-month treatment and 6-month treatment, respectively. Pearson et al. (2007) [58] defined response to sulfonylurea as failure to reach a target HbA1c < 7% within 1-year treatment and minimum HbA1c achieved within 1-year treatment, and they also considered time taken on sulfonylurea treatment to achieve target HbA1c < 7% as a drug response phenotype. In addition, Sesti et al. (2006) [45] defined secondary sulfonylurea failure as FPG greater than 300 mg/dl despite sulfonylurea-metformin combined therapy and appropriate diet, in the absence of other conditions causing hyperglycemia, but Holstein et al. (2011) [60] defined secondary sulfonylurea failure as the addition of insulin after at least 6-month sulfonylurea therapy and corresponding HbA1c ≥ 7%. In the following, we first summarize major results of SNPs’ effects on sulfonylurea responses in a gene-by-gene manner, and then, we present functional prediction results for nsSNPs and non-coding SNPs by respective online bioinformatics tools.

**Table 1:** Characteristics of included studies (*N* = 17)^*^

Study ID	Author	Year	Gene Symbol	SNP Name	dbSNP ID	Study Design	Study Subjects	Control Source	Length of Follow-up	Results	Association
Study 1	Gloyn et al. [44]	2001	*KCNJ11*	E23K	rs5219	RCT	363 Caucasian T2D and 307 normoglycemic control subjects	UKPDS	1 year	Variant allele did not significantly affect the response to SU therapy significantly	No
Study 2	Sesti et al. [45]	2006	*KCNJ11*	E23K	rs5219	RCT	525 Caucasian T2D patients with secondary SU failure	Hospital-based	NA	Secondary SU failure, K allele vs E allele (OR = 1.45; 95% CI 1.01–2.09; *P* = 0.04). Adjustment for age, gender, fasting glycemia, glycosylated hemoglobin, age at diagnosis, and duration of diabetes in a logistic regression analysis did not change this association (OR = 1.69; 95% CI: 1.02–2.78; *P* = 0.04)	Yes
Study 3	Feng et al. [46]	2008	*KCNJ11*	E23K	rs5219	RCT	1268 Chinese T2D patients treated with 8-week gliclazide	Hospital-based	8 weeks	E23K variant of the *KCNJ11* gene was significantly associated with decreases in FPG (P = 0.002).	Yes
Study 4	Holstein et al. [47]	2009	*KCNJ11*	E23K	rs5219	Case–control	43 T2D patients treated with glimepiride or glibenclamide	Hospital-based	NA	E23K variant was significantly associated with increased HbA1c levels (adjusted *P* = 0.04) independent of age, sex, body mass index, diabetes duration and SU dose.	Yes
Study 5	Nikolac et al. [48]	2009	*KCNJ11*	E23K	rs5219	Cross-sectional	228 Caucasian T2D patients with SU therapy	Hospital-based	NA	For KCNI11 E23K polymorphism, for different genotype groups, there were no significant differences of FPG, PPG, and HbA1c concentrations (*P* = 0.143, 0.675, and 0.824, respectively)	No
Study 6	El-sisi et al. [49]	2011	*KCNJ11*	E23K	rs5219	Case–control	50 Egyptian T2D patients with secondary SU failure	Hospital-based	NA	Secondary SU failure, EK + KK vs. EE (RR = 1.65; 95% CI: 1.04–2.6; *P* = 0.04).	Yes
Study 7	Javorsky et al. [50]	2012	*KCNJ11*	E23K	rs5219	RCT	55 T2D patients with 6-month treatment of gliclazide	Hospital-based	6 months	For ΔHbA1c EK + KK vs. EE (1.15 ± 0.09 vs. 0.80 ± 0.13, *P* = 0.036)	Yes
Study 7	Javorsky et al. [50]	2012	*KCNJ11*	E23K	rs5219	RCT	28 T2D patients with 6-month treatment of glimepiride	Hospital-based	6 months	For ΔHbA1c EK + KK vs. EE (1.10 ± 0.12 vs. 1.00 ± 0.19 *P* = 0.676)	No
Study 7	Javorsky et al. [50]	2012	*KCNJ11*	E23K	rs5219	RCT	14 T2D patients with 6-month treatment of glibenclamide	Hospital-based	6 months	For ΔHbA1c EK + KK vs. EE (1.05 ± 0.11 vs. 0.98 ± 0.09 *P* = 0.633)	No
Study 8	Ragia et al. [51]	2012	*KCNJ11*	E23K	rs5219	Case–control	92 T2D patients (80 glimepiride/12 gliclazide) who had experienced at least one drug-associated hypoglycemic event, while 84 T2D patients (74 glimepiride/10 gliclazide) who had never experienced a hypoglycemic event	Hospital-based	NA	*KCNJ11* E23K genotype and allele frequencies were not different between hypoglycemic and non-hypoglycemic T2D patients (*P* = 0.35 and 0.47, respectively). In logistic regression models before and after adjustment for other risk factors (age, body mass index, sulfonylurea mean daily dose, duration of T2D, renal function and CYP2C9 genotype), *KCNJ11* E23K polymorphism did not affect hypoglycemia risk	No
Study 9	Li et al. [52]	2014	*KCNJ11*	E23K	rs5219	RCT	108 Chinese T2D patients treated with gliclazide for 16 weeks	Hospital-based	16 weeks	Patients with the KK genotype had larger augmentations in changes (Δ) in acute insulin response (*P* = 0.049) and D body mass index (*P* = 0.003); Patients with the EK genotype had a lower variance in changes in fasting insulin levels (*P* = 0.049) and homeostasis model assessment of β cell function (*P* = 0.021) than those with the KK genotype	Yes
Study 1	Gloyn et al. [44]	2001	*KCNJ11*	L270V	rs1800467	RCT	363 Caucasian T2D patients	UKPDS	1 year	Variant allele did not significantly affect the response to SU therapy significantly	No
Study 10	Meirhaeghe et al. [53]	2001	*ABCC8*	Intron 15, exon 16 -3C/T	rs1799854	Cross-sectional	70 T2D patients with SU therapy	3 large representative French samples (in Lille, Strasbourg, and Toulouse) participating in the risk factor surveys of the WHO-MONICA	NA	For T2D patients treated with SU agents, those subjects bearing at least one -3C allele and had fasting plasma TG concentrations 35% lower than TT homozygotes [2.20 mmol/L (1.14–4.14) for TT vs. 1.43 mmol/L (0.81–2.52) for TC + CC; *P* = 0.026]	Yes
Study 11	Zychma et al. [54]	2002	*ABCC8*	Intron 15, exon 16 -3C/T	rs1799854	Case–control	68 Caucasian T2D patients who required insulin treatment and had known diabetes duration ≤ 5 years, compared to 99 Caucasian T2D patients receiving SU alone or in combination with metformin or acarbose with known diabetes duration ≥ 15 years	Hospital-based	NA	There was no significant impact of *ABCC8* exon 16 -3C/T polymorphism on the early ineffectiveness of SU treatment (*P* = 0.4126 based on a Chi-square test)	No
Study 5	Nikolac et al. [48]	2009	*ABCC8*	Intron 15, exon 16 -3C/T	rs1799854	Cross-sectional	228 Caucasian T2D patients with SU therapy	Hospital-based	NA	CC genotype of the *ABCC8* exon 16 polymorphism had significantly lower HbA1c concentration compared to the patients with T genotype [6.9 (6.2–7.7) mmol/L vs. 8.1 (6.7–8.8) mmol/L; *P* = 0.009]	Yes
Study 12	Nikolac et al. [55]	2012	*ABCC8*	Intron 15, exon 16 -3C/T	rs1799854	Cross-sectional	251 Caucasian T2D patients with SU therapy	Hospital-based	NA	Polymorphic allele carriers of the *ABCC8* intron 15 -3C/T (which is 3 bp ahead of exon 16) polymorphism were more frequent in the subgroup of patients with the TG concentration increase after 6 months (P for genotype and allelic differences: 0.024 and 0.015, respectively)	Yes
Study 13	Zhang et al. [56]	2007	*ABCC8*	A1369S	rs757110	RCT	115 T2D patients with gliclazide treatment for 8 weeks	Hospital-based	8 weeks	For ΔHbA1c TG + GG vs. TT (1.60 ± 1.39 vs. 0.76 ± 1.70, *P* = 0.044)	Yes
Study 3	Feng et al. [46]	2008	*ABCC8*	A1369S	rs757110	RCT	1268 Chinese T2D patients treated with 8-week gliclazide	Hospital-based	8 weeks	Compared with TT genotype, subjects with the GG genotype had a 7.7% greater decrease in FPG (P < 0.001), an 11.9% greater decrease in 2-h plasma glucose (*P* = 0.003), and a 3.5% greater decrease in HbA1c (*P* = 0.06)	Yes
Study 14	Sato et al. [57]	2010	*ABCC8*	A1369S	rs757110	Case–control	32 patients with T2D admitted to hospital with severe hypoglycemia and 125 consecutive T2D outpatients without severe hypoglycemia, and all of the patients were taking glimepiride or glibenclamide	Hospital-based	NA	There were no significant differences in *ABCC8* A1369S genotype distribution between patients with or without severe hypoglycemia (*P* = 0.26). Moreover, the A1369 allele tended to be less frequent in the hypoglycemic group (31 vs. 43%; OR = 1.65; 95% CI: 0.92–2.96; *P* = 0.09)	No
Study 5	Nikolac et al. [48]	2009	*ABCC8*	R1273R^**^	rs1799859	Cross-sectional	228 Caucasian T2D patients with SU therapy	Hospital-based	NA	GG genotype of the *ABCC8* exon 31 polymorphism had significantly higher HbA1c concentration compared to the AA genotype [7.8 (6.9–8.8) mmol/L vs. 6.3 (5.7–6.8) mmol/L; P < 0.001]	Yes
Study 12	Nikolac et al. [55]	2012	*ABCC8*	R1273R^**^	rs1799859	Cross-sectional	251 Caucasian T2D patients with SU therapy	Hospital-based	NA	Wile-type G allele carriers had a significantly higher TG concentration when compared with the carriers of two variant A alleles (*P* = 0.023)	Yes
Study 15	Pearson et al. [58]	2007	*TCF7L2*	NA	rs7903146	RCT	901 T2D patients with SU treatment	GoDARTS	12 months	Carriers of the risk allele were less likely to respond to SUs with an OR for failure of 1.95 (95% CI: 1.23–3.06; *P* = 0.005), comparing rs12255372 TT vs. GG. Including the baseline HbA1c strengthened this association (OR = 2.16, 95% CI: 1.21–3.86, *P* = 0.009)	Yes
Study 16	Schroner et al. [59]	2011	*TCF7L2*	NA	rs7903146	RCT	87 T2D patients with 6-month SU treatment in addition to metformin	Hospital-based	6 months	Reduction in HbA1c: CC vs. CT + TT is 1.16 ± 0.07 vs. 0.86 ± 0.07%, *P* = 0.003 Reduction in FPG: 1.57 ± 0.12 vs. 1.14 ± 0.14 mmol/L, P = 0.031)	Yes
Study 17	Holstein et al. [60]	2011	*TCF7L2*	NA	rs7903146	RCT	189 T2D patients with 6-month SU treatment	Hospital-based	6 months	T allele was significantly more frequent in the group of patients who failed to respond to SU (i.e., those with HbA1c ≥ 7%) (36%) than in the control (i.e., those with HbA1c < 7%) group (26%) (OR = 1.57, 95% CI: 1.01-2.45, *P* = 0.046)	Yes

^*^Studies are grouped by different genes. For each gene, studies are first sorted by SNP Name, then by Year, and then by Author, in ascending orders. *Abbreviations*: *CI* confidence interval, *FPG* fasting plasma glucose, *Go-DARTS* Genetics of Diabetes Audit and Research Study in Tayside Scotland, *HbA1c* glycated hemoglobin A1c, *OR* odds ratio, *RCT* randomized clinical trial, *SNP* single nucleotide polymorphism, *SU* sulfonylurea, *SUR* sulfonylurea receptor, *T2D* type 2 diabetes, *TG* triglyceride, *UKPDS* United Kingdom Prospective Diabetes Study, *WHO-MONICA* World Health Organization-Multinational MONItoring of trends and determinants of CArdiovascular diseases, *NA* not available

^**^Because R1273R is a synonymous SNP, it is not included in functional prediction

### *KCNJ11*

The most widely studied genetic polymorphism of *KCNJ11* for sulfonylurea response is E23K (i.e., rs5219) located in exon 1 [33]. However, functional effects of *KCNJ11* E23K polymorphism on the secretion and sensitivity of insulin in humans remain contentious [5]. Recent larger studies demonstrated that a significant reduction of insulin secretion, lower levels of insulin, and an improvement of insulin sensitivity were related to E23K variant in *KCNJ11* gene [61]. Moreover, E23K variant was associated with T2D development, which means that the K allele carriers had an increased risk of T2D [44, 62, 63]. Furthermore, some studies also found that the K allele carriers had better therapeutic response to gliclazide in comparison with the EE homozygous wild-type group [50], as well as an increased risk of sulfonylurea treatment failure [45, 49]. In addition, E23K variant was significantly associated with an increase of glycated hemoglobin A1c (HbA1c) level [47] and fasting glucose level that patients with the KK homozygous variant genotype had lower fasting glucose levels than those with the EE/EK heterozygous genotype [52]. Importantly, recent evidence demonstrated that patients with *KCNJ11* variants responded more efficiently to sulfonylurea than insulin [64–66]. Another *KCNJ11* polymorphism that was associated with sulfonylurea treatment responses is rs5210 which is located in 3’- untranslated region (UTR). A study conducted in two independent cohorts of Chinese T2D patients (cohort 1: *n* = 661, cohort 2: *n* = 607) treated with gliclazide demonstrated that *KCNJ11* rs5210 was positively associated with gliclazide response in cohort 1 study [46].

### *ABCC8*

The most widely studied genetic polymorphism of *ABCC8* for sulfonylurea response is S1369A (i.e., rs757110) located in exon 33 [67]. This genetic variant was demonstrated to influence antidiabetic efficacy of sulfonylurea treatment in Chinese [46, 56], as well as an increased sensitivity to gliclazide [56]. More importantly, *KCNJ11* E23K and *ABCC8* S1369A, two common K_ATP_ channel mutations that were in strong linkage disequilibrium, form a haplotype that appears to be associated with an increased T2D risk [68]. Additional *ABCC8* gene polymorphisms including rs1799854 (intron 15, exon 16 -3C/T) and rs1799859 (exon 31) had been shown to be associated with sulfonylurea treatment efficacy in Caucasians [48, 55].

### *TCF7L2*

Previous studies have shown that several non-coding genetic variants of *TCF7L2* are associated with T2D risk in populations of diverse ancestries from countries encompassing United Kingdom [69], the Netherlands [70], Finland [32], Sweden [71], France [72], United States [73], India [74], and Japan [75] populations. Among these T2D-associated *TCF7L2* variants, rs7903146 (intron 4) showed the strongest association with T2D [76]. Significant reductions in HbA1c and fasting plasma glucose levels following a combined sulfonylurea and metformin treatment between T2D patients with CC genotype and those with CT/TT genotype were associated with *TCF7L2* rs7903146 variant allele [59]. Moreover, the rs12255372 variant, together with the rs7903146 variant, was shown to be associated with a significantly more frequent treatment failure [58–60]. It shall be noted that although in previous literatures, e.g., as in [32, 77], *TCF7L2* rs7901695 and rs7903146 are indicated to be in intron 3, and rs7895340, rs11196205 and rs12255372 are indicated to be in intron 4, this is because exon 4, which is a variable exon, is often named as “3a” [78]. Because of a high incorporation in pancreatic β cells [79], exon 4 shall be included in the gene structure, such that rs7901695 and rs7903146 shall be indicated as located in intron 4, and rs7895340, rs11196205, and rs12255372 in intron 5, respectively, e.g., as in [80]. For the linear ordering of these 5 non-coding SNPs, according to the most updated (i.e., as of April 18, 2017) NCBI dbSNP, the chromosomal coordinates for rs7901695, rs7903146, rs7895340, rs11196205 and rs12255372 are 112994329, 112998590, 113041766, 113047288, and 113049143, respectively, on human chromosome 10 based on GRCh38.p7 assembly. Therefore, the linear ordering shall be rs7901695-rs7903146-rs7895340-rs11196205-rs12255372, as shown in Fig. 4 (all drawings in Figs. 1, 2, 3, and 4 are not to their exact scales and are for illustration purposes), which is agreement with that of [77].

#### *In silico* bioinformatics analysis results

For *KCNJ11*, *ABCC8* and *TCF7L2* genes, functional prediction results for 36 nsSNPs by SIFT, PolyPhen-2, PANTHER, MutPred, and SNPs3D were presented in Table 2, and those prediction results for 7 non-coding SNPs by HaploReg v4.1, RegulomeDB and Ensembl’s VEP were presented in Table 3.

**Table 2:** *In silico* predicted functional effects of 36 non-synonymous SNPs in the pharmacogenetics of sulfonylureas treatment by SIFT, PolyPhen-2, PANTHER, MutPred, and SNPs3D*

SNP ID	Gene Symbol	SNP Name	dbSNP ID	SNP Location	Chromosome Location (GRCh38.p7)	SIFT Score/Prediction	PolyPhen-2 Score/Prediction	PANTHER subSPEC	PANTHER P_deleterious_	MutPred P_deleterious_	SNPs3D Score
SNP1	*KCNJ11*	E23K	rs5219	Exon 1	11:17388025	1.00/Tolerated	0.001/Benign	−0.69172	0.09044	0.35	2
SNP2	*KCNJ11*	R27H	NA	Exon 1	NA	0.18/Tolerated	0.006/Benign	−3.75303	0.67984	0.248	NA
SNP3	*KCNJ11*	V59M	NA	Exon 1	NA	0.12/Tolerated	0.999/Probably damaging	−2.72126	0.43076	0.855	NA
SNP4	*KCNJ11*	I182V	NA	Exon 1	NA	0.98/Tolerated	0.998/Probably damaging	−1.62168	0.20128	0.684	NA
SNP5	*KCNJ11*	R192H	NA	Exon 1	NA	0.01/Affect Protein Function	1.000/Probably damaging	−6.9765	0.98159	0.816	NA
SNP6	*KCNJ11*	R201H	rs80356624	Exon 1	11:17387490	0.00/Affect Protein Function	1.000/Probably damaging	NA	NA	0.981	NA
SNP7	*KCNJ11*	E227K	NA	Exon 1	NA	0.00/Affect Protein Function	1.000/Probably damaging	−7.17583	0.98487	0.94	NA
SNP8	*KCNJ11*	L270V	rs1800467	Exon 1	11:17387284	0.13/Tolerated	0.003/Benign	−1.54301	0.18893	0.566	0.68
SNP9	*KCNJ11*	I337V	rs5215	Exon 1	11:17387083	0.73/Tolerated	0.000/Benign	−0.89045	0.10817	0.462	0.94
SNP10	*KCNJ11*	S385C	rs41282930	NA	11:17386938	0.02/Affect Protein Function	0.380/Possibly damaging	NA	NA	0.229	NA
SNP11	*ABCC8*	G7R	NA	Exon 1	NA	0.00/Affect Protein Function	1.000/Probably damaging	NA	NA	0.863	NA
SNP12	*ABCC8*	N24K	NA	Exon 1	NA	0.03/Affect Protein Function	1.000/Probably damaging	NA	NA	0.877	NA
SNP13	*ABCC8*	F27S	NA	Exon 1	NA	0.00/Affect Protein Function	0.884/Probably damaging	NA	NA	0.858	NA
SNP14	*ABCC8*	N72S	rs80356634	Exon 2	11:17474961	0.12/Tolerated	0.402/Possibly damaging	NA	NA	0.802	NA
SNP15	*ABCC8*	R74W	NA	Exon 2	NA	0.00/Affect Protein Function	1.000/Probably damaging	NA	NA	0.904	NA
SNP16	*ABCC8*	A116P	NA	NA	NA	0.12/Tolerated	1.000/Probably damaging	NA	NA	0.825	NA
SNP17	*ABCC8*	E128K	NA	Exon 3	NA	0.02/Affect Protein Function	1.000/Probably damaging	NA	NA	0.829	NA
SNP18	*ABCC8*	F132L	rs80356637	Exon 3	11:17470119	0.16/Tolerated	0.877/Possibly damaging	NA	NA	0.847	NA
SNP19	*ABCC8*	V187D	NA	Exon 4	NA	0.01/Affect Protein Function	0.042/Benign	NA	NA	0.857	NA
SNP20	*ABCC8*	L213R	rs80356642	Exon 5	11:17461767	0.41/Tolerated	0.212/Possibly damaging	−3.12006	0.52998	0.786	NA
SNP21	*ABCC8*	E382K	rs80356651	NA	11:17453151	0.27/Tolerated	0.392/Possibly damaging	−1.96296	0.26172	0.872	NA
SNP22	*ABCC8*	R495Q	NA	Exon 10	NA	0.00/Affect Protein Function	1.000/Probably damaging	−8.28432	0.99496	0.906	NA
SNP23	*ABCC8*	E501K	NA	Exon 10	NA	0.00/Affect Protein Function	1.000/Probably damaging	−2.39817	0.35392	0.948	NA
SNP24	*ABCC8*	L503P	NA	Exon 10	NA	0.00/Affect Protein Function	1.000/Probably damaging	−4.69708	0.84515	0.964	NA
SNP25	*ABCC8*	F686S	NA	Exon 15	NA	0.01/Affect Protein Function	0.998/Probably damaging	−4.1174	0.75351	0.909	NA
SNP26	*ABCC8*	G716V	rs72559723	Exon 16	11:17427124	0.18/Tolerated	1.000/Probably damaging	−8.97797	0.99747	0.974	NA
SNP27	*ABCC8*	K1336N	NA	NA	NA	0.25/Tolerated	0.016/Benign	−1.84974	0.24044	0.693	NA
SNP28	*ABCC8*	L1349Q	NA	Exon 33	NA	0.01/Affect Protein Function	0.997/Probably damaging	−4.16681	0.76257	0.912	NA
SNP29	*ABCC8*	A1369S	rs757110	Exon 33	11:17396930	0.51/Tolerated	0.000/Benign	−0.85285	0.1046	0.323	0.87
SNP30	*ABCC8*	S1386F	NA	Exon 34	NA	0.00/Affect Protein Function	1.000/Probably damaging	−4.60703	0.833	0.95	NA
SNP31	*ABCC8*	L1389P	NA	Exon 34	NA	0.00/Affect Protein Function	0.993/Probably damaging	−4.93634	0.87395	0.886	NA
SNP32	*ABCC8*	R1420C	rs28938469	Exon 35	11:17395659	0.00/Affect Protein Function	1.000/Probably damaging	NA	NA	0.863	−1.19
SNP33	*ABCC8*	I1424V	rs80356653	Exon 35	11:17395647	0.00/Affect Protein Function	0.988/Probably damaging	−2.02622	0.27413	0.882	NA
SNP34	*ABCC8*	D1471H	NA	Exon 36	NA	0.00/Affect Protein Function	0.994/Probably damaging	−4.60764	0.83308	0.913	NA
SNP35	*TCF7L2*	P179H	rs3197486	NA	10:113141236	0.00/Affect Protein Function	1.000/Probably damaging	−5.62868	0.93268	0.879	0.01
SNP36	*TCF7L2*	K323N	rs2757884	NA	10:113151761	0.00/Affect Protein Function	1.000/Probably damaging	−4.23841	0.77529	0.309	−0.15

^*^ *Abbreviations*: *MutPred* Mutation Prediction, *PANTHER*, Protein ANalysis THrough Evolutionary Relationships, *PolyPhen-2* Polymorphism Phenotyping v2, *SIFT* Sorting Intolerant from Tolerant, *SNP* Single Nucleotide Polymorphism, *subSPEC* subStitution Position-specific Evolutionary Conservation, *NA* Not Available

**Table 3:** *In silico* predicted functional effects of 7 non-coding SNPs in the pharmacogenetics of sulfonylureas treatment by Haploreg v4.1, RegulomeDB, and Ensembl’s VEP*

SNP ID	Gene Symbol	dbSNP ID	SNP Location	Chromosome Location (GRCh38.p7)	HaploReg v4.1 Motifs changed by SNP	RegulomeDB Score/Prediction	Ensembl’s VEP
SNP37	*KCNJ11*	rs5210	3’-UTR	11:17386704	None	4/Minimal binding evidence	NA
SNP38	*ABCC8*	rs1799854	Intron 15	11:17427157	4 altered motifs	5/Minimal binding evidence	NA
SNP39	*TCF7L2*	rs7895340	Intron 5	10:113041766	Irf, PRDM1	NA	NA
SNP40	*TCF7L2*	rs7901695	Intron 4	10:112994329	None	5/Minimal binding evidence	NA
SNP41	*TCF7L2*	rs7903146	Intron 4	10:112998590	7 altered motifs	5/Minimal binding evidence	Risk factor
SNP42	*TCF7L2*	rs11196205	Intron 5	10:113047288	SMC3	5/Minimal binding evidence	Risk factor
SNP43	*TCF7L2*	rs12255372	Intron 5	10:113049143	5 altered motifs	NA	Risk factor

*Abbreviations: *RegulomeDB* Regulome Database, *SNP* Single Nucleotide Polymorphism, *UTR* Untranslated Region, *VEP* Variant Effect Predictor, *NA* Not Available

##### Analysis of functional effects of SNPs by SIFT

SIFT was used to predict the functional impact of an nsSNP on a protein molecule. An nsSNP with a SIFT score ≤ 0.05 is considered as having a deleterious effect on protein function [81]. A total of 22 nsSNPs were predicted to affect protein function (SIFT score range: 0.00-0.03) including 4 *KCNJ11* missense SNPs (R192H, R201H, E227K, S385C), 16 *ABCC8* missense SNPs (G7R, N24K, F27S, R74W, E128K, V187D, R495Q, E501K, L503P, F686S, L1349Q, S1386F, L1389P, R1420C, I1424V, D1471H), and 2 *TCF7L2* missense SNPs (P179H, K323N), whereas the remaining 14 missense SNPs were predicted to be tolerated (SIFT score range: 0.12–1.00) (Table 2).

##### Analysis of functional effects of nsSNPs by PolyPhen-2

PolyPhen-2 calculates a naïve Bayes posterior probability for a given mutation that it will be benign (PolyPhen-2 score < 0.15), possibly damaging (PolyPhen-2 score is greater than or equal to 0.15 but is less than 0.85), or probably damaging (PolyPhen-2 score ≥ 0.85), respectively [82]. A total of 25 nsSNPs were predicted to be probably damaging to protein function (PolyPhen-2 score range: 0.877–1.000), which includes 5 *KCNJ11* missense SNPs (V59M, I182V, R192H, R201H, E227K), 18 *ABCC8* missense SNPs (G7R, N24K, F27S, R74W, A116P, E128K, F132L, R495Q, E501K, L503P, F686S, G716V, L1349Q, S1386F, L1389P, R1420C, I1424V, D1471H) and 2 *TCF7L2* missense SNPs (P179H, K323N), and the remaining 11 SNPs were classified as benign (PolyPhen-2 score range: 0.000–0.402) (Table 2).

##### Analysis of functional effects of nsSNPs by PANTHER

PANTHER characterizes likely functional effect of amino acid variation by means of a hidden Markov model-based statistical modeling and evolutionary relationship. The SNP with subSPEC score ≤ −3 is considered as intolerant or deleterious, whereas SNP with subSPEC score > −3 is classified to be less deleterious [83]. A total of 14 amino acid substitutions were classified as intolerant (subSPEC score range: from−8.97797 to−3.12006) including 3 *KCNJ11* missense SNPs (R27H, R192H, E227K), 9 *ABCC8* missense SNPs (L213R, R495Q, L503P, F686S, G716V, L1349Q, S1386F, L1389P, D1471H) and 2 *TCF7L2* missense SNPs (P179H, K323N), another 10 amino acid substitutions were classified as tolerated (subSPEC score range: from−2.72126 to−0.69172), and the remaining 12 amino acid substitutions did not have subSPEC scores (Table 2).

##### Analysis of functional effects of nsSNPs by MutPred

MutPred predicts molecular causes of disease or deleterious amino acid substitution. A total of 30 nsSNPs had *p*-values > 0.5, which were considered to be functional [84] (MutPred P_deleterious_ range: 0.566-0.981), which included 6 *KCNJ11* missense SNPs (V59M, I182V, R192H, R201H, E227K, L270V), 23 *ABCC8* missense SNPs (G7R, N24K, F27S, N72S, R74W, A116P, E128K, F132L, V187D, L213R, E382K, R495Q, E501K, L503P, F686S, G716V, K1336N, L1349Q, S1386F, L1389P, R1420C, I1424V, D1471H) and 2 *TCF7L2* missense SNPs (P179H, K323N) (Table 2).

##### Analysis of functional consequences of SNPs by SNPs3D

SNPs3D assigns molecular functional effects of nsSNPs based on structure and sequence analysis. Of the 36 nsSNPs, SNPs3D SVM score was available for only 7 nsSNPs (*KCNJ11*: 2, *ABCC8*: 3, and *TCF7L2*: 2). Of them, two nsSNPs, i.e., R1420C amino acid substitution of *ABCC8* gene and K323N amino acid substitution of *TCF7L2* gene, had SVM scores < 0, which were classified as deleterious substitutions [85] (Table 2).

##### Analysis of functional consequences of SNPs by HaploReg v4.1

HaploReg v4.1 is an online software for exploring annotations of the non-coding genome among those results of published genome-wide association studies or new sets of genetic variants, which help researchers to integrate DNA regulatory elements data with genetic variants to quickly formulate novel biological hypotheses [40, 41]. As predicted by HaploReg v4.1, rs1799854, rs7895340, rs7903146, rs11196205 and rs12255372 could change 4, 2 (i.e., Irf and PRDM1), 7, 1 (i.e., SMC3), and 5 DNA motifs for DNA-binding proteins, and could have regulatory effects on gene transcription. Neither rs5210 nor rs7901695 appear to change known motifs (Table 3).

##### Analysis of functional consequences of SNPs by RegulomeDB

RegulomeDB is a database that annotates SNPs with known and predicted regulatory elements in the intergenic regions of the human genome. Of the 7 non-coding SNPs, rs5210, rs1799854, rs7901695, rs7903146, and rs11196205 had RegulomeDB scores of 4, 5, 5, 5, and 5, respectively, which were all classified as having minimal binding evidence. Predictions were not available for either rs7895340 or rs12255372 (Table 3).

##### Analysis of functional consequences of SNPs by Ensembl’s VEP

The Ensembl’s VEP determines the effects of genetic variants on genes, transcripts, and protein sequences, as well as regulatory regions. Three non-coding SNPs of *TCF7L2* gene, i.e., rs7903146, rs11196205 and rs12255372, were predicted as risk factors (Table 3).

## Discussion

Sulfonylureas are a class of drugs that stimulates insulin secretion by closing K_ATP_ channels in pancreatic β cells. It has been estimated that 10–20% of individuals treated do not attain adequate glycemic control, and 5–10% initially responding to sulfonylurea subsequently lose the ability to maintain near-normal glycemic level [86]. This implies that genetic factors are linked with treatment efficacy of sulfonylureas. In our study, that includes 17 studies, two *KCNJ11* SNPs — rs5219 (E23K) (exon 1) and rs5210 (3’-UTR), three *ABCC8* SNPs — rs757110 (A1369S) (exon 33), rs1799854 (intron 15, exon 16 -3C/T), rs1799859 (R1273R) (exon 31), and two *TCF7L2* SNPs rs7903146 (intron 4) and rs12255372 (intron 5) have been associated with response to sulfonylureas. Based on bioinformatics predictions for 36 selected coding SNPs (all are missense) for *KCNJ11*, *ABCC8*, and *TCF7L2*, by applying a set of computational tools — SIFT, PolyPhen-2, PANTHER, MutPred, and SNPs3D. Our bioinformatics prediction results demonstrated that 8 *KCNJ11* missense SNPs (R27H, V59M, I182V, R192H, R201H, E227K, L270V, and S385C), 23 *ABCC8* missense SNPs (G7R, N24K, F27S, N72S, R74W, A116P, E128K, F132L, V187D, L213R, E382K, R495Q, E501K, L503P, F686S, G716V, K1336N, L1349Q, S1386F, L1389P, R1420C, I1424V, D1471H), and 2 *TCF7L2* missense SNPs (P179H, K323N) could affect protein functions with SIFT score ≤ 0.05, or PolyPhen-2 score ≥ 0.85, or PANTHER subSPEC score ≤ −3, or MutPred > 0.5, or SNPs3D score < 0. Of them, previous studies showed that mutant alleles of 4 *KCNJ11* missense SNPs (R27H, V59M, R192H, and R201H) and 5 *ABCC8* missense SNPs (G7R, N24K, F27S, R74W, and E128K) can be successfully rescued by sulfonylurea treatments. In addition, 3 *TCF7L2* non-coding SNPs — rs7903146, rs11196205 and rs12255372 were predicted as risk factor based on Ensembl’s VEP, although their functional impacts in sulfonylurea results need to be elucidated by further experimental studies.

## Conclusion

The ultimate goal of pharmacogenetics is the development of personalized medicine through individual genetic profiles which would accurately predict which individuals with a specific medical condition would respond to a specific medical therapy. Traditional medicine refers to the broad application of “standard of care” or “one-size-fits-all” treatments to all patients with a given diagnosis. In contrast, personalized medicine, often described as providing “the right drug for the right patient at the right dose and time” [87], tailors medical treatment according to each patient’s personal history, genetic profile and/or specific biomarkers [88, 89], Therefore, the full application of personalized medicine in health care will require significant changes in regulatory and reimbursement policies as well as legislative protections for privacy. The U.S. Food and Drug Administration has updated the labels of more than 120 drugs with recommendations for genetic testing prior to their use [90]. Currently, most genetic testing is based genotypic effects. Haplotypes of multiple linked genetic variants provide more precise information of their functional impacts than individual genetic markers [91, 92], which could also be potentially important for diagnosis and prognosis [93]. In future, regulatory authorities shall formulate clear guidelines for evaluating and approving personalized diagnostics and therapeutics and identify patients who can benefit from them.

## Abbreviations

ABCC8: ATP Binding Cassette Subfamily C Member 8
ADP: Adenosine diphosphate
ATP: Adenosine triphosphate
cAMP: Cyclic adenosine monophosphate
CL: cytosolic loop
dbSNP: Single Nucleotide Polymorphism database
EMBASE: Excerpta Medica Database
GLP-1: Glucagon-like peptide-1
HbA1c: Glycated hemoglobin A1c
Go-DARTS: Genetics of Diabetes Audit and Research Study in Tayside Scotland
K_ATP_: ATP-sensitive K^+^ channel
KCNJ11: Potassium channel, inwardly rectifying subfamily J, member 11
Kir: Inwardly rectifying K(+)
LEF: Lymphocyte enhancer factor
MutPred: Mutation prediction
NBD: nucleotide binding domain
NCBI: National Center for Biotechnology Information
nsSNP: Non-synonymous single nucleotide polymorphism
PANTHER: Protein analysis through evolutionary relationships
PKA: Protein kinase A
PolyPhen-2: Polymorphism phenotyping v2
RCT: Randomized clinical trial
RegulomeDB: Regulome database
SIFT: Sorting intolerant from tolerant
SNP: Single nucleotide polymorphism
SU: Sulfonylurea
subSPEC: subStitution Position-specific Evolutionary Conservation
SUR: Sulfonylurea receptor
T2D: Type 2 diabetes
TCF: T-cell factor
TCF7L2: T-cell factor 7-like 2
TMD: transmembrane domain
UKPDS: United Kingdom Prospective Diabetes Study
UTR: Untranslated region
VEP: Variant effect predictor
WHO-MONICA: World Health Organization-Multinational MONItoring of trends and determinants of CArdiovascular diseases
Wnt: Wingless type
